# Patient Factors Associated With Surgical Outpatient Non-attendance in a Remote Australian Context

**DOI:** 10.7759/cureus.104773

**Published:** 2026-03-06

**Authors:** Zachary K Woodward, Nariyoshi Miyata, Eric Owusu, Manprit Kaur, Grace Lloyd, T'Kido Titasey, Francis Asomah

**Affiliations:** 1 Department of Surgery, Mount Isa Hospital, Mount Isa, AUS

**Keywords:** aboriginal and torres strait islander, outpatient clinic, patient non-attendance, regional australia, surgical outpatient clinic

## Abstract

Background

Non-attendance at outpatient clinics compromises continuity of care, clinic efficiency, and health equity. In Australia, these challenges are amplified in remote settings and among Aboriginal and Torres Strait Islander peoples, yet data on surgical outpatient attendance in such contexts are limited.

Methods

A retrospective cohort study was conducted of all adult patients scheduled for appointments at a surgical outpatient clinic in Mount Isa, Queensland, Australia, between January 1, 2022, and December 31, 2024. Demographic, geographic, and appointment-related variables were analyzed. Geographic access was assessed using collapsed Modified Monash Model (MMM) categories and distance to clinic. Univariable logistic regression identified predictors of non-attendance, followed by multivariable logistic regression to estimate adjusted associations. Two multivariable models were constructed, incorporating either the MMM category or the distance.

Results

A total of 6,267 appointments for 2,792 patients were analyzed, with an overall non-attendance rate of 18.3%. In multivariable analysis, Indigenous status was the strongest independent predictor for non-attendance (adjusted OR 3.72, 95% CI 3.22-4.29, p < 0.001). Increasing age was associated with improved attendance (adjusted OR 0.97 per year, p < 0.001). Geographic remoteness and greater travel distance were both associated with higher odds of non-attendance. Telephone (adjusted OR 0.57, 95% CI 0.45-0.71, p < 0.001) and telehealth consultations (adjusted OR 0.62, 95% CI 0.47-0.82, p = 0.001) were associated with reduced non-attendance after adjustment. Female gender showed a reversal from higher unadjusted odds to lower adjusted odds of non-attendance.

Conclusion

Non-attendance at surgical outpatient clinics in remote Australia is common and is associated with Indigenous status, geographic access, age, and appointment modality. Systemic and logistical barriers to healthcare access continue to disproportionately affect Indigenous patients and those residing further from care, resulting in an unequal burden of missed appointments. Telehealth and telephone consultations are associated with improved attendance and represent important tools that may help mitigate geographic barriers.

## Introduction

Outpatient clinic attendance plays a crucial role in ensuring timely diagnosis, management, and continuity of care across a range of surgical conditions. However, missed appointments remain a persistent challenge, particularly in geographically remote and socioeconomically disadvantaged communities. Missed appointments also lead to an inefficient use of resources, imposing substantial financial costs on the healthcare system and increasing wait times for patients [1]. 

Aboriginal and Torres Strait Islander peoples are distinct cultural groups, with Aboriginal peoples originating from the mainland of Australia and Torres Strait Islander peoples originating from the Torres Strait Islands between northern Queensland and Papua New Guinea. For Aboriginal and Torres Strait Islander peoples (hereafter respectfully referred to as Indigenous peoples), the challenge of patient non-attendance is further compounded by longstanding disparities in healthcare access and outcomes between Indigenous and non-Indigenous Australians. Indigenous Australians continue to experience significantly poorer health outcomes, including higher morbidity and mortality, compared with their non-Indigenous counterparts [2].

Mount Isa, a remote Australian community in northwest Queensland, reflects these disparities acutely, with Indigenous peoples comprising 21.5% of the local population, substantially higher than the national average of 3.2% [3]. Previous research has demonstrated lower rates of outpatient attendance among Indigenous Australians [4,5]. Despite these findings, limited data exist regarding surgical outpatient attendance specifically in remote settings, where logistical, cultural, and systemic barriers may be particularly pronounced. One study identified several potential contributors to reduced engagement of Indigenous patients in healthcare, including competing family and kinship obligations, feelings of shame or embarrassment, suboptimal communication from healthcare staff, and challenges navigating complex health system structures [6].

This study aimed to investigate the demographic factors associated with non-attendance at surgical outpatient clinics in a remote Australian context, with a focus on identifying patient factors that are associated with missed appointments. By analyzing retrospective data from a three-year period, this research seeks to elucidate patterns of non-attendance and inform strategies to improve engagement with surgical services in remote and underserved populations.

## Materials and methods

A retrospective cohort analysis was performed of all patients with a scheduled appointment at the surgical outpatient department between January 1, 2022, and December 31, 2024. The data utilized in this study were obtained from routinely collected administrative records for the surgical outpatient department.

The Mount Isa surgical outpatient clinic conducts two morning sessions per week, held on Wednesdays and Fridays. These sessions comprise a combination of in-person consultations, telephone reviews, and telehealth video appointments. Patients are allocated 20-minute appointment slots. Each clinic typically reviews approximately 20-25 patients, including 12-16 in-person consultations, 4-6 telephone appointments, and 2-3 telehealth consultations.

In addition to SMS reminders, patients are contacted by telephone on the day prior to their scheduled appointment to confirm the details. For patients without access to a reliable phone service, appointment letters are either mailed to their residential address or, where feasible, hand-delivered locally. Where required, transport is arranged to facilitate attendance. Outpatient consultations are provided free at the point of care, with no direct financial cost to the patient.

Referrals are triaged according to clinical urgency, with Category 1, 2, and 3 patients targeted to be seen within 30, 90, and 365 days, respectively. However, due to the relatively short waitlist, even Category 3 patients are typically reviewed within eight weeks of referral.

The policy for patients who do not attend their clinic appointment is that, in the first instance, they are rebooked for the next available session or for an alternative appointment time at the patient’s convenience. A patient who has failed to attend two consecutive appointments is removed from the waitlist, and their referrer is notified; however, in some instances, a patient may be offered additional appointments at the discretion of the clinician. Patients who cancel or reschedule their appointment are not counted as non-attenders. 

Inclusion criteria/exclusion criteria

All patients over the age of 18 who were scheduled to have an in-person consult, phone review, or telehealth appointment at the Mount Isa Hospital surgical outpatient department from January 1, 2022, to December 31, 2024, were included. A total of nine patients were excluded from the final dataset due to incomplete medical records.

Analysis

Descriptive statistics were used to compare attenders and non-attenders for age, gender, Indigenous status, and type of appointment. Geographical remoteness was classified according to the Modified Monash Model (MMM) [7] and collapsed into the following four categories to reduce sparse data bias and improve interpretability: MMM 6, large regional center; MMM 1-2, metropolitan/inner regional; MMM 3-5, rural; and MMM 7, very remote. Distance from the surgical clinic was calculated in a straight-line from the suburb the patient resides in to Mount Isa and was analyzed both as a continuous variable and categorized into four groups (<50 km, 50-199 km, 200-499 km, ≥500 km) for descriptive and univariable analyses.

Each factor was regressed with univariable logistic regression for the outcome of non-attendance; odds ratios (ORs) and 95% confidence intervals (CIs) are reported. Statistically significant factors were further analyzed in multivariable analysis. Two multivariable logistic regression models were constructed to identify factors independently associated with non-attendance. The primary multivariable model included age, gender, Indigenous status, appointment modality, and collapsed MMM categories. The secondary multivariable model replaced MMM with continuous distance to clinic. Distance and MMM were not included in the same multivariable model due to collinearity between these measures of geographic access. Reference categories for regression analysis were in-person consultations, non-Indigenous status, and male gender. As Mount Isa is classified as a large regional center (MMM 6), this category was used as the reference group for analysis. Year 2024 was selected as the reference category because it had the largest sample size and was temporally more distant from the COVID-19 pandemic. Country of birth was excluded from univariate and multivariable analyses due to sparse category counts and overlap with primary demographic predictors. For all analyses, a p-value of <0.05 was considered statistically significant. Statistical analysis was performed with IBM SPSS Statistics for Windows, Version 26.0 (Released 2018; IBM Corp., Armonk, NY, USA).

## Results

The demographic information for patients seen at the outpatient clinic is provided in Table 1. A total of 6,267 outpatient appointments were scheduled for 2,792 patients between January 1, 2022, and December 31, 2024. Of them, 3,472 appointments (55.4%) were for males, and 2,795 appointments (44.6%) were for females. The age ranged from 18 to 99 years, with an average of 54.2 years (SD ± 17.2).

**Table 1:** Patient demographic information and type of appointment. Percentages are presented as a proportion of total appointments across 2022-2024. MMM: Modified Monash Model.

	Total	2022	2023	2024
Appointments	6,267 (100.0%)	1,629 (26.0%)	1,904 (30.4%)	2,734 (43.6%)
In-person	4,836 (77.2%)	1,299 (20.7%)	1,515 (24.2%)	2,022 (32.3%)
Phone	932 (14.9%)	202 (3.2%)	194 (3.1%)	536 (8.6%)
Telehealth	499 (8.0%)	128 (2.0%)	195 (3.1%)	176 (2.8%)
Age				
Average age ± SD	54.2 ± 17.1	53.6 ± 17.7	53.84 ± 17.4	54.8 ± 16.7
Gender				
Male	3,472 (55.4%)	951 (15.2%)	1,023 (16.3%)	1,498 (23.9%)
Female	2,795 (44.6%)	678 (10.8%)	881 (14.1%)	1,236 (19.7%)
Indigenous status				
Non-Indigenous	4,314 (68.8%)	1,141 (18.2%)	1,282 (20.5%)	1,891 (30.2%)
Indigenous	1,953 (31.2%)	488 (7.8%)	622 (9.9%)	843 (13.5%)
Aboriginal	1,827 (29.2%)	470 (7.5%)	584 (9.3%)	773 (12.3%)
Torres Strait Islander	41 (0.7%)	5 (0.1%)	19 (0.3%)	17 (0.3%)
Both Aboriginal and Torres Strait Islander	85 (1.4%)	13 (0.2%)	19 (0.3%)	53 (0.8%)
Remoteness - MMM				
MMM 1-2: metropolitan/inner regional	99 (1.6%)	31 (0.5%)	44 (0.7%)	24 (0.4%)
MMM 3-5: rural	40 (0.6%)	15 (0.2%)	15 (0.2%)	10 (0.2%)
MMM 6: large regional center	4,923 (78.6%)	1,266 (20.2%)	1,456 (23.2%)	2,201 (35.1%)
MMM 7: very remote	1,205 (19.2%)	317 (5.1%)	389 (6.2%)	499 (8.0%)
Country of origin				
Australia	5,252 (83.8%)	1,374 (21.9%)	1,579 (25.2%)	2,299 (36.7%)
New Zealand	249 (4.0%)	56 (0.9%)	83 (1.3%)	110 (1.8%)
England	163 (2.6%)	40 (0.6%)	52 (0.8%)	71 (1.1%)
Philippines	122 (1.9%)	28 (0.4%)	31 (0.5%)	63 (1.0%)
Other	481 (7.7%)	131 (2.1%)	159 (2.5%)	191 (3.0%)

The mean number of appointments per patient over the three-year period was 2.24 (SD ± 1.87), with the highest number recorded for a single patient being 24 appointments. Across appointment modalities, 4,836 were in-person consultations, 932 were telephone appointments, and 499 were telehealth video consultations. The most common country of origin was Australia (83.8%), followed by New Zealand (4.0%), England (2.6%), the Philippines (1.9%), and numerous other countries, each of which constituted less than a percentage. Regarding Indigenous status, 4,314 (68.8%) appointments were made for non-Indigenous patients and 1,953 (31.2%) appointments for Indigenous patients.

The overall non-attendance rate was 18.3%. When stratified by appointment type, the non-attendance rate for in-person appointments was 19.8%, compared with 11.4% for telephone and 16.8% for telehealth appointments.

Univariate analysis

Univariable associations between demographic, geographic, and appointment-related factors and non-attendance are shown in Table 2. In univariable logistic regression, compared with in-person consultations, phone appointments were associated with significantly lower odds of non-attendance (OR 0.520, 95% CI 0.420-0.644, p < 0.001), while there was no significant difference for telehealth video appointments.

**Table 2:** Attendance and non-attendance rates with unadjusted odds ratios with 95% confidence intervals from univariate logistic regression for demographic, geographic, and appointment-related factors. MMM: Modified Monash Model.

	Attended	Non-attended	Odds ratio (95% CI)	p-value
Gender				
Male	2,874 (82.8%)	598 (17.2%)	Reference	-
Female	2,246 (80.4%)	549 (19.6%)	1.175 (1.033-1.336)	0.014
Age				
Per 1-year increase	-	-	0.968 (0.964-0.971)	<0.001
Indigenous status				
Non-Indigenous	3,831 (88.8%)	483 (11.2%)	Reference	
Indigenous	1,289 (66.0%)	664 (34.0%)	4.086 (3.577-4.668)	<0.001
Appointment type				
In-person	3,879 (80.2%)	957 (19.8%)	Reference	-
Phone	826 (88.3%)	106 (11.4%)	0.520 (0.420-0.644)	<0.001
Telehealth	415 (83.2%)	84 (16.8%)	0.820 (0.642-1.048)	0.113
Year				
2024	2,369 (86.6%)	365 (13.4%)	Reference	
2023	1,482 (77.8%)	422 (22.2%)	1.848 (1.584-2.157)	<0.001
2022	1,269 (77.9%)	360 (22.1%)	1.841 (1.568-2.162)	<0.001
MMM category				
MMM 6: large regional center	4,095 (83.2%)	828 (16.8%)	Reference	-
MMM 1-2: metropolitan/inner regional	65 (65.7%)	34 (34.3%)	2.587 (1.697-3.943)	<0.001
MMM 3-5: rural	27 (67.5%)	13 (32.5%)	2.381 (1.224-4.634)	0.011
MMM 7: very remote	933 (77.4%)	272 (22.6%)	1.442 (1.24–1.682)	<0.001
Distance				
Per 1-km increase	-	-	1.001 (1.001-1.001)	<0.001
<50 km	4,086 (83.2%)	824 (16.8%)	Reference	-
50-199 km	379 (82.4%)	81 (17.6%)	1.060 (0.824-1.363)	0.651
200-499 km	545 (75.0%)	182 (25.0%)	1.656 (1.378-1.990)	<0.001
>500 km	110 (64.7%)	60 (35.3%)	2.705 (1.958-3.737)	<0.001

Female patients showed a 17.5% increased risk of non-attendance to all appointment types with an OR of 1.175 (95% CI 1.033-1.336). Increasing age was strongly associated with improved attendance, with each one-year increase associated with a 3.2% reduction in the odds of non-attendance (OR 0.968, 95% CI 0.964-0.971, p < 0.001).

While Indigenous patients comprised 31.2% of the outpatient population, they accounted for 58.0% of all non-attended appointments. Indigenous status was the strongest independent predictor of non-attendance and was associated with more than fourfold higher odds of non-attendance (OR 4.086, 95% CI 3.577-4.668, p < 0.001).

Geographic remoteness, measured using collapsed MMM categories, was significantly associated with non-attendance. Compared with patients attending from large regional centers, those from metropolitan/inner regional areas (OR 2.587, 95% CI 1.697-3.943, p < 0.001), rural areas (OR 2.381, 95% CI 1.224-4.634, p = 0.011), and very remote areas (OR 1.44, 95% CI 1.24-1.68, p < 0.001) all demonstrated higher odds of non-attendance. However, the sample sizes for metropolitan and rural categories were relatively small, and the corresponding confidence intervals were wide, warranting cautious interpretation of these estimates.

Distance to the clinic was also strongly associated with non-attendance. When analyzed as a continuous variable, increasing distance was associated with progressively higher odds of non-attendance (OR 1.001 per km, p < 0.001). When categorized, no significant difference was observed for patients residing 50-199 km from the clinic compared with those living within 50 km. However, patients residing 200-499 km away (OR 1.66, 95% CI 1.38-1.99, p < 0.001) and those living ≥500 km away (OR 2.71, 95% CI 1.96-3.74, p < 0.001) had substantially higher odds of non-attendance.

Multivariable analysis

A multivariable logistic regression model was fitted to estimate the adjusted effects of age, geographic access, gender, Indigenous status, and appointment modality on non-attendance. The model demonstrated good overall fit (χ²(10) = 763.768, p < 0.001), with Cox and Snell and Nagelkerke pseudo-R² values of 0.115 and 0.187, respectively, which indicates that the model explains approximately 11.5%-18.7% of the variance in non-attendance. The results of the multivariable analysis are shown in Table 3.

**Table 3:** Multivariable logistic regression models for non-attendance with adjusted odds ratios and 95% confidence intervals. Primary model included appointment modality, gender, age, Indigenous status, and MMM category, while the secondary model substituted MMM with continuous distance to clinic. MMM: Modified Monash Model.

	Primary model OR (95% CI)	p-value	Secondary model OR (95% CI)	p-value
Gender				
Male	Reference	-	Reference	
Female	0.850 (0.738-0.979)	0.024	0.846 (0.735-0.974)	0.020
Age				
Per 1-year increase	0.972 (0.968-0.976)	<0.001	0.972 (0.968-0.976)	<0.001
Indigenous status				
Non-Indigenous	Reference	-	Reference	
Indigenous	3.719 (3.223-4.291)	<0.001	3.702 (3.216-4.261)	<0.001
Appointment type				
In-person	Reference	-	Reference	
Phone	0.567 (0.453-0.710)	<0.001	0.618 (0.493-0.775)	<0.001
Telehealth	0.617 (0.467-0.815)	<0.001	0.601 (0.462-0.782)	<0.001
Year				
2024	Reference	-		
2023	1.786 (1.514-2.106)	<0.001	1.780 (1.509-2.100)	<0.001
2022	1.824 (1.536-2.166)	<0.001	1.834 (1.544-2.178)	<0.001
MMM category				
MMM 6: large regional center	Reference	-	-	-
MMM 1-2: metropolitan/inner regional	2.598 (1.638-4.120)	<0.001	-	-
MMM 3-5: rural	3.645 (1.809-7.347)	<0.001	-	-
MMM 7: very remote	1.263 (1.054-1.514)	0.011	-	-
Distance				
Per 1-km increase	-	-	1.001 (1.001-1.001)	<0.001

Increasing age remained a strong protective factor, with each one-year increase associated with a 2.8% reduction in the odds of non-attendance (adjusted OR 0.972, 95% CI 0.968-0.976, p < 0.001). Indigenous status remained the strongest independent predictor, with Indigenous patients demonstrating more than threefold higher odds of non-attendance compared with non-Indigenous patients (adjusted OR 3.719, 95% CI 3.223-4.291, p < 0.001).

For appointment modality, telephone appointments remained associated with reduced odds of non-attendance after adjustment (adjusted OR 0.567, 95% CI 0.453-0.710, p < 0.001). Telehealth video consultations were also associated with significantly lower odds of non-attendance after adjustment (adjusted OR 0.617, 95% CI 0.467-0.815, p = 0.001), in contrast to the univariate analysis.

Geographic remoteness remained independently associated with non-attendance. Compared with patients attending from large regional centers, those residing in metropolitan/inner regional areas (adjusted OR 2.598, 95% CI 1.638-4.120), rural areas (adjusted OR 3.645, 95% CI 1.809-7.347), and very remote areas (adjusted OR 1.263, 95% CI 1.054-1.514) all demonstrated higher odds of non-attendance after multivariable adjustment.

In contrast to the univariate analysis, multivariable analysis demonstrated that female gender was associated with slightly lower odds of non-attendance (adjusted OR 0.850, 95% CI 0.738-0.979, p = 0.017).

In a secondary multivariable model in which the MMM was replaced with continuous distance to clinic, increasing travel distance remained independently associated with non-attendance (adjusted OR 1.001 per kilometer, 95% CI 1.001-1.001, p < 0.001). Age, Indigenous status, gender, and appointment modality demonstrated similar effect sizes and directions to those observed in the primary model, suggesting that geographic remoteness and travel distance capture related but distinct dimensions of access to care.

## Discussion

Overall, non-attendance affected 18.3% of scheduled visits, which is notably higher than rates reported in comparable studies where non-attendance has typically ranged from 4.9% to 10.9% [4,5,8]. This discrepancy may in part reflect the remote setting of the present study, in contrast to the metropolitan or rural contexts of prior work, where logistical and structural barriers to attendance are typically less pronounced.

Indigenous status emerged as the strongest independent predictor of non-attendance, and although they represented less than one-third of the clinic population, Indigenous patients accounted for more than half of all missed outpatient appointments. They also demonstrated more than threefold higher odds of missing appointments after adjustment for age, gender, appointment modality, year, and geographic access. Two previous Australian studies investigating attendance at general practitioner and urology outpatient clinics reported odds ratios of 3.0 and 2.8 for Indigenous patient non-attendance, respectively [5,8]. While this result is similar to the 3.7 OR found in this study, it should be noted that the prior studies were conducted in rural and metropolitan contexts where Indigenous people comprise a substantially lower proportion of the population.

An additional study from Queensland of dermatology outpatient appointments at an inner regional center reported an Indigenous non-attendance rate of 25.9% [4], which is lower than the 34.0% observed in this study. Unfortunately, ORs were not calculated in that study, which limits direct comparison.

Identified issues within the healthcare system, which contribute to Indigenous non-attendance, include inadequate communication within the healthcare system, poor health literacy, socioeconomic disadvantage, transport limitations, competing social and family responsibilities, and cultural barriers [8,9]. 

Geographic remoteness and travel distance were both independently associated with higher odds of non-attendance, a finding consistent with the published literature [5]. Very remote areas were significantly more likely to miss appointments compared with those from large regional centers, and increasing distance remained predictive of non-attendance when modelled as a continuous variable. Although patients from metropolitan/inner regional and rural areas demonstrated poorer outpatient attendance, these findings should be interpreted with caution due to small sample sizes within these categories. Due to Mount Isa’s remote location situated far from metropolitan centers, and relatively transient population, it is likely some patients returned home and were subsequently unable to attend their scheduled outpatient appointments.

Both telephone and telehealth video consultations were associated with significantly lower odds of non-attendance after adjustment, with telehealth showing a protective effect that was not evident in univariate analysis. This reversal suggests that unadjusted comparisons were confounded by demographic and access-related factors, including age, Indigenous status, and geographic location. Once these factors were accounted for, remote modalities appeared to facilitate attendance rather than hinder it. Despite supportive findings from previous studies for remote consultations [10,11], a statewide Queensland analysis of specialist outpatient appointments found no difference between non-attendance for telehealth appointments compared with in-person consultations and identified forgetfulness or confusion about appointment details as stronger contributors to non-attendance than travel distance [12]. While telehealth has a role in reducing non-attendance rates, future efforts to increase attendance, which prioritize improved patient communication, may be more effective than reducing travel burden.

Difficulty contacting patients is a key mechanism by which socioeconomic disadvantage contributes to non-attendance. A Queensland Government audit found that 60% of patients who were not contactable failed to attend their outpatient appointments [13], a finding that aligns with the challenges observed in this remote context, where many of the most vulnerable patients lack a fixed address or reliable access to telephone communication.

Increasing age was associated with improved attendance, which is consistent with previous studies, likely reflecting greater health literacy, prioritization of medical care, and fewer competing work or caregiving responsibilities among older patients [5,8,12,14-16]. In contrast, female gender was associated with higher odds of non-attendance in univariate analysis but lower odds after multivariable adjustment, indicating that the apparent gender differences were confounded by other factors such as age, geographic access, and appointment modality. The existing literature regarding the influence of gender on outpatient non-attendance is conflicting, with some studies reporting higher non-attendance among males or females, while others demonstrate no significant association [5,8,14,15]. It is also plausible that attendance patterns vary across subspecialties, which may partially account for these inconsistent findings.

The findings of this study have important implications for the design of outpatient services, particularly in remote and underserved communities. While the issue of outpatient non-attendance is multifaceted, this study demonstrates the need for the implementation of targeted strategies to improve engagement, particularly among Indigenous patients. Further research should focus on elucidating the systemic and logistical barriers that prevent patients from attending outpatient appointments, thereby enabling the development of patient-centered initiatives to reduce non-attendance. For Indigenous patients, such strategies should be developed in partnership with Indigenous stakeholders to ensure they are tailored to community needs and aligned with culturally safe models of care.

## Conclusions

Non-attendance at surgical outpatient clinics in remote Australia is common and is associated with Indigenous status, geographic access, younger age, and appointment modality. Indigenous patients and those residing further from care experience a disproportionate burden of missed appointments, reflecting enduring structural inequities in access to healthcare. Addressing non-attendance in remote surgical services is a complex challenge that will require sustained, coordinated action to achieve meaningful improvement. Future efforts should prioritize improving engagement among the most vulnerable communities, with a specific focus on enhancing access to and engagement with Indigenous Australians. Finally, the observed benefits of telephone and video consultations support their ongoing integration into surgical outpatient practice where appropriate.
